# A rapid assessment of post-disclosure experiences of urban HIV-positive and HIV-negative school-aged children in Kenya

**DOI:** 10.7717/peerj.956

**Published:** 2015-06-11

**Authors:** Grace Gachanja

**Affiliations:** College of Health Sciences, Walden University, Minneapolis, MN, USA

**Keywords:** Psychological effects of HIV disclosure, HIV disclosure, HIV/AIDS, Resource-poor nation, Qualitative research, Parent HIV status disclosure, Kenya, Child HIV status disclosure

## Abstract

There has been limited involvement of HIV-negative children in HIV disclosure studies; most studies conducted on the effects of disclosure on children have been with HIV-positive children and HIV-positive mother-child dyads. Seven HIV-positive and five HIV-negative children participated in a larger study conducted to understand the lived experiences of HIV-positive parents and their children during the disclosure process in Kenya. In this study, the experiences of these 12 children after receiving disclosure of their own and their parents’ illnesses respectively are presented. Each child underwent an in-depth qualitative semi-structured digitally recorded interview. The recorded interviews were transcribed and loaded into NVivo8 for phenomenological data analysis. Five themes emerged from the data, indicating that HIV-positive and negative children appear to have differing post-disclosure experiences revolving around acceptance of illness, stigma and discrimination, medication consumption, sexual awareness, and use of coping mechanisms. Following disclosure, HIV-negative children accepted their parents’ illnesses within a few hours to a few weeks; HIV-positive children took weeks to months to accept their own illnesses. HIV-negative children knew of high levels of stigma and discrimination within the community; HIV-positive children reported experiencing indirect incidences of stigma and discrimination. HIV-negative children wanted their parents to take their medications, stay healthy, and pay their school fees so they could have a better life in the future; HIV-positive children viewed medication consumption as an ordeal necessary to keep them healthy. HIV-negative children wanted their parents to speak to them about sexual-related matters; HIV-positive children had lingering questions about relationships, use of condoms, marriage, and childbearing options. All but one preadolescent HIV-positive child had self-identified a person to speak with for social support. When feeling overwhelmed by their circumstances, the children self-withdrew and performed positive activities (e.g., praying, watching TV, listening to the radio, singing, dancing) to help themselves feel better. Many HIV-affected families have a combination of HIV-positive and negative siblings within the household. Pending further studies conducted with larger sample sizes, the results of this study should assist healthcare professionals to better facilitate disclosure between HIV-positive parents and their children of mixed HIV statuses.

## Introduction

HIV/AIDS remains a public health issue affecting 35.3 million persons globally (UNAIDS, 2013). In 2012, 22% of Kenya’s population were children between 10 and 19 years old (UNAIDS, 2013). The HIV prevalence among children aged 18 months to 14 years was 0.9% (National AIDS and STI Control Programme Kenya, 2014) and 2.7% among youth aged 15–24 years (UNICEF, 2013). In 2012, the adult HIV prevalence was 6% (UNAIDS, 2013), and 5% of Kenyan homes had a HIV-positive head of household (National AIDS and STI Control Programme Kenya, 2014).

Following disclosure, HIV-positive and negative children are known to experience varying effects (Kennedy et al., 2010; Murphy, 2008; Vallerand et al., 2005). After disclosure of their illnesses, HIV-positive teenage children in Puerto Rico went through the five stages of grieving (denial, anger, bargaining, depression, and acceptance) before accepting their illnesses (Blasini et al., 2004). In studies conducted in the United States using mother-child dyads, HIV-negative children were reported as faring no worse after receiving disclosure of their mothers’ illnesses (Jones et al., 2007; Murphy, Steers & Stritto, 2001; Shafer et al., 2001). HIV-positive mothers in a South African study, reported that their HIV-negative children accepted disclosure of their illnesses calmly; however, some showed emotions such as surprise and confusion (Rochat et al., 2014; Rochat, Mkwanazi & Bland, 2013).

Some positive effects of disclosure on HIV-positive and negative children include increased closeness with their parents (Vallerand et al., 2005), fewer behavioral problems and aggression (Lee & Rotheram-Borus, 2002; Murphy, Steers & Stritto, 2001), and improved resiliency, coping, and life perspectives (Kennedy et al., 2010; Murphy et al., 2010). Negative internalized effects of disclosure include poor functioning, increased stress, sadness, withdrawal, depression, and fear (Asander et al., 2009; Kennedy et al., 2010; Murphy, 2008; Petersen et al., 2010; Vallerand et al., 2005; Wiener et al., 2007). Negative externalized effects of disclosure include arguing with or ignoring parents, aggression, and practicing unsafe sexual behavior (Lee & Rotheram-Borus, 2002; Murphy, 2008; Nelms & Zeigler, 2008; Vallerand et al., 2005).

High levels of HIV stigma and discrimination are known to exist in Kenya (Gachanja, Burkholder & Ferraro, 2014a; Gachanja, Burkholder & Ferraro, 2014b; Turan et al., 2012). Stigma is experienced externally as felt stigma when the HIV-positive or HIV-affected person experiences bullying, teasing, insults, gossip, and ostracism; or internally when he or she perceives him or herself as unworthy due to discriminative acts or stigmatizing behavior directed towards him or her by his or her community members (Ishikawa et al., 2010; Petersen et al., 2010). Midtbo et al. (2012) reported that HIV-positive children in their study conducted in Botswana and Tanzania experienced stigma from their peers and community members; however, most confidently took control of their illnesses without negatively internalizing their experiences. There have been few studies involving HIV-negative children; therefore, their stigma-related experiences in relation to disclosure of their parents’ illnesses are not well documented.

The stress and coping theory was used as the foundation for this study (Lazarus, 1993). Coping is assessed by how well a person cognitively and behaviorally addresses the stress he or she experiences. The theory posits that stress management involves problem- or emotion-focused coping strategies, and that there is no universal good or bad way to cope with stress. A person’s problem-focused coping is enhanced by self-adaptation to his or her environment, while emotion-focused coping is improved by being hypervigilant and anticipative of which situations lead to stress, and then avoiding those stressors (Lazarus, 1993). Assessing a person’s thoughts and coping behaviors is important because improved handling of stressors helps him or her understand, handle, and lessen the stress associated with his or her unchangeable circumstances (Lazarus, 1993).

It is not well understood if HIV-positive and negative children experience similar effects following disclosure of their own and their parents’ illnesses respectively. Most studies on disclosure to children conducted in Sub-Saharan Africa have reported on the effects of disclosure on HIV-positive children after being told about their own illnesses (Brown et al., 2011; Menon et al., 2007; Petersen et al., 2010; Vaz et al., 2010). A few recent studies have reported on the effects of maternal disclosure on preadolescent HIV-negative children (Rochat et al., 2014; Rochat, Mkwanazi & Bland, 2013). A larger study was conducted to understand the lived experiences of HIV-positive parents and their children during the HIV disclosure process in Kenya; seven HIV-positive and five HIV-negative children participated in that study. Data previously reported from this child sample conveyed these children’s views on how HIV disclosure should be approached and performed to children (Gachanja, Burkholder & Ferraro, 2014a). In this current study, the post-disclosure experiences of these 12 children are presented to add to the body of knowledge on the effects of disclosure on HIV-positive and negative children after they receive disclosure of their own and their parents’ illnesses respectively.

## Methods

### Recruitment of participants

Data collection for the larger study was conducted in December 2010 through January 2011 at the Kenyatta National Hospital Comprehensive Care Center located in Nairobi, Kenya. Participant recruitment for the larger study was continued until interview data saturation was achieved (Kuzel, 1999; Morse, 2000), upon which recruitment was halted resulting in a child sample size of seven HIV-positive and five HIV-negative children (Gachanja, Burkholder & Ferraro, 2014a). The HIV-positive and negative children were purposively selected to be in the study because they were between 8 and 17 years old, conversant in English, and had already received partial or full disclosure of their own and their parents’ illnesses respectively. Ethics approval for the study was received from the Kenyatta National Hospital Research Standards and Ethics Committee (Approval # P373/10/2010) and the Walden University Institutional Review Board (Approval # 11-10-10-03904).

HIV-positive parents who had HIV-negative children meeting criteria for study participation were approached during their regularly scheduled clinic visits, provided with an explanation of the study, and requested to bring their HIV-negative children to the clinic at a time convenient to them. HIV-positive children who met criteria to be in the study were approached for participation during their regularly scheduled clinic visits. An explanation of the study was provided to them and their parents. HIV-positive and negative children who expressed an interest to participate in the study were escorted by the researcher (accompanied by their parents) to a private room in the clinic where consenting and study procedures were performed. Following consenting procedures, those children who agreed to participate provided written assent and their parents provided written informed consent.

### Data collection

Qualitative interpretive phenomenological data was collected through in-depth individualized semi-structured interviews conducted with each child by the researcher. Interview guides used in the study were in English and had been obtained for use with permission from the authors of a study conducted in the Democratic Republic of Congo (Vaz et al., 2008). The guides were not translated into a local Kenyan language because only children conversant in English were purposively recruited into the study. HIV-positive children were interviewed on their experiences about receiving disclosure of their own illnesses, and HIV-negative children were interviewed on their experiences about receiving disclosure of their parents’ illnesses.

The interview guide questions collected basic child demographic information, and also explored how and who had disclosed to the children, their reactions to disclosure, and their experiences following disclosure. Parents were given the option of being present in the room during their children’s interview sessions; however, none chose to do so and all children assented to being interviewed alone. Interviews lasted between 30 and 45 minutes; however, one HIV-negative child did not finish his entire interview because he became very emotional when describing his disclosure experiences. He was referred to the psychologist’s office for counseling and follow up.

### Data analysis

Recorded interviews were transcribed soon after data collection by the researcher and a local Kenyan university student experienced with transcription. The transcripts were checked twice against the recorded interviews for accuracy and loaded into NVivo8 for analysis. The Van Kaam method (Moustakas, 1994) was used for phenomenological analysis of the transcribed qualitative data. Transcripts were listed, grouped, and scanned repeatedly for emerging codes. Repeating information within the transcripts was clustered into similar codes. The codes and themes were cross-checked by the research committee for coding reliability and consistency within each emerging theme. The codes were then grouped into five emergent themes describing the children’s post-disclosure experiences.

## Results

**Figure 1:**
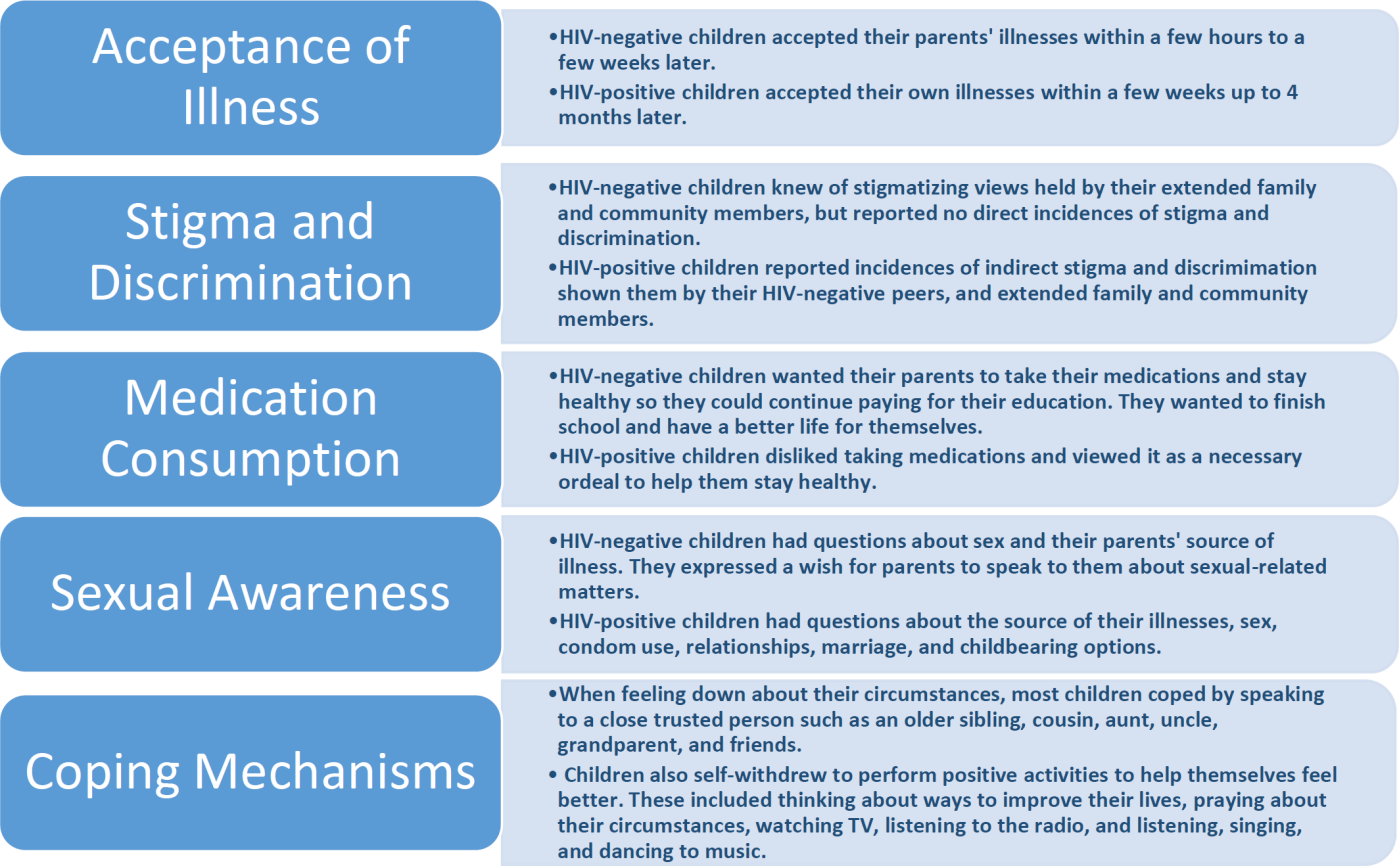
HIV-positive and HIV-negative children’s post-disclosure themes.

The 12 children’s demographic characteristics are displayed in Table 1. Six HIV-positive children had full disclosure of their illnesses, and three HIV-negative children had full disclosure of their parents’ illnesses. All HIV-positive children were taking antiretroviral therapy, multivitamins, and cotrimoxazole; all HIV-negative children were aware that their parents consumed medications on a daily basis. The five themes which emerged from the data include acceptance of illness, stigma and discrimination, medication consumption, sexual awareness, and coping mechanisms; they are displayed in Fig. 1 and further described below. The key quotes from each theme are displayed in Table 2.

**Table 1:** Sample demographic characteristics.

Variable	Frequency	
	HIV-positive children	HIV-negative children
**Age**		
12–13	2	1
14–15	1	3
16–17	4	1
**Gender**		
Female	3	3
Male	4	2
**Educational status**		
Primary	2	3
Secondary	5	2
**HIV disclosure status**		
Partial disclosure	1	2
Full disclosure	6	3

**Table 2:** Key quotes from each theme.

Theme	HIV-positive children	HIV-negative children
**Acceptance of illness**	**Quote 1** HIV-positive girl: [Clicks tongue] I was hopeless, [clicks tongue] I hated myself, [clicks tongue] even I almost lost hope in life…I came to counselling and the counselor taught me how to take medicine, and the consequences [clicks tongue]…I used to cry then after sometime maybe like two months that’s when [clicks tongue] I started accepting myself. Now [clicks tongue] I feel just like a normal human being, I just take it like a cold…But I still blame my dad coz he knew he was positive yet he let my mother give birth to me and my mother never knew she had the disease. [Tongue clicks during conversations in Kenya depict discomfort with the topic being discussed].	**Quote 2** HIV-negative girl: [After partial disclosure] I felt relieved because I knew that God will protect her [mother], and maybe she could go on well and get better…I wanted to know of my mother’s ailment so that I can assist her in any way that I can, by doing the work that she needs to be done, like working in the house and feeding the chicken.
**Stigma and discrimination**	**Quote 3** HIV-positive boy: The way people talk about AIDS sometimes I don’t like it, about the medicine, the ARVs! I think when you are thin they say you are positive but when you are fat they say you are not. They say so many things about HIV positive people that I don’t like…You see in school when you have HIV status, many people joke, they say if I know about my HIV status, I can kill myself, I don’t know what, and then I cannot take those drugs, meaning you feel very badly.	**Quote 4** HIV-negative boy: If you are HIV-positive, then you sometimes are an outcast. They [mother’s in-laws] say that you have been witchcrafted, so telling them it will be gossip news, they will be talking about it every time. Even my brother does not know why she [mother] takes the drugs, I don’t know if my father knows, but she told me he does not know. They all know that because she was sick with meningitis, she takes medicine ever since. I prefer not to tell him, it is not because of hatred or something, it is because if I tell my brother he will go and tell my father.
**Medication consumption**	**Quote 5** HIV-positive girl: Sometimes when I have stress as in I am being shouted at, I just sit down and start crying and other stuff. I ask myself questions which I cannot answer by myself; so actually there is nobody who can help me. I usually ask myself, why was it supposed to be me? Why is it me who is supposed to take all these drugs all the time? Why is it me I am the only HIV-positive girl in the house? I never used to take drugs, but now I have to stick on them [sighs] until [pauses, hits table], until this world comes to an end. I am a kid, now you know I have stress all the time, thinking I am the only person who has all these diseases, I am taking all these drugs [sighs]. Actually I hate taking drugs all the time, actually it sucks coz usually my brother and sisters just go to bed, me I have to take medicine before I go to bed [sighs], and in the morning the same thing.	**Quote 6** HIV-negative boy: She [mother] got sick and stayed at the hospital for about three months. She is better now, she usually comes here, takes her medicine and then goes back home. Even sometimes when she forgets to take her medicine, because she always takes her medicine at eight am and eight pm, I remind her [laughs].
**Sexual awareness**	**Quote 7** HIV-positive boy: [Researcher: Do you have any questions that you have wanted to ask?] Yes, when I am positive and I decide to get married can I get a child who is negative and I cannot transfer the disease to my wife? [Have you asked anybody that question?] Yeah. [Who did you ask?] I asked a psychologist here in the CCC. [Did she answer your question?] She told me, I can’t remember exactly what, but your male sperm is taken to the lab and they are treated, then they are taken and transferred to your wife and she gets pregnant without the disease. [Do you feel that she answered your questions completely?] No, it is still in my mind. [Even after she answered your question?] Yeah. [Why is it still in your mind?] What about if it is done physically as in the ordinary way [without a condom]? The way she told me it is very expensive, what about if you cannot afford it, what can you do?	**Quote 8** HIV-negative girl: We are very close, I can just see something or hear something and I ask her [mother]. She never hides anything from me. If it is about sex, she tells me this goes on, and you know about even condoms and not most parents are usually open with their children…My mum disclosed to me and that’s a great thing because now I know this AIDS thing is real, it is there, so it’s like she is telling me to be careful myself not to end up the way she has. So I think it is best for kids to know about it.
**Coping mechanisms**	**Quote 9** HIV-positive boy: I speak to my friends when we come here for our club when the schools are closed. There are many of us, we come and play together and we share experiences like how we felt when we were first told. We discuss about our lives, how we are living, how we should live, how we should take our medicine, how we should eat, and how we should control ourselves…[HIV-positive children] need to receive care, be loved, and to be shown they are just like other people, like they be respected not when they come to the hospital they are told you wait because you are HIV. No, they be treated like the other people... They should be encouraged to do their favorite things, you know you can’t force me to do something [chores] I don’t like, even that one will make me idle.	**Quote 10** HIV-negative boy: When I got to know her status, I told my friend [whose mother is HIV-positive] that my mother is positive. Then he encouraged me that I just take things as normal…I think [HIV-affected] children should be encouraged to get together because if I am a friend then you tell me that your mother is HIV-positive since this age, then I recently know that my father or my mother is HIV-positive, you could help me to know how to take care of him or her or even how to take it positive that she is sick. Some people just see it as a very big sickness that can kill somebody or kill the parent, so it is better to talk to your friend, you speak out what you are feeling.

### Acceptance of illness

Regardless of the type of disclosure received, 11 of 12 children were shocked at the time of disclosure, but expressed they were happy to have been disclosed to. Now that they knew of the illness, one HIV-positive girl and an HIV-negative boy with full disclosure did not want to be frequently reminded of the illness. After their shock wore off, most of the six HIV-positive children with full disclosure became depressed, received counseling, accepted their illnesses, and returned to “normal” anywhere from a few weeks up to four months later. Two of these HIV-positive children still expressed blame and anger at their parents for infecting them (see Table 2, Quote 1). HIV-negative children (with partial and full disclosure) overcame their shock and accepted their parents’ illnesses within a few hours to a few weeks later; none received counseling. Most explained they grew closer to their parents, were empathetic about their illnesses, and helped out more with chores to ease their parents’ burden of illness (see Table 2, Quote 2).

### Stigma and discrimination

Both HIV-positive and negative children were aware of high stigma and discrimination levels prevalent in the community; some expressed their siblings and close relatives did not know of theirs and their parents’ illness respectively. HIV-positive children spoke of misconceptions and incidences of indirect stigma and discrimination shown them by their HIV-negative peers, and extended family and other community members (see Table 2, Quote 3). HIV-negative children, including those with partial disclosure, were secretive and protective of their parents’ illnesses. Those with full disclosure expressed awareness of discriminative views held against HIV-positive persons by their extended family and other community members (see Table 2, Quote 4). As a result of stigma, HIV-positive and negative children generally hid theirs and their parents’ illnesses respectively from others.

### Medication consumption

Medication consumption was a way of life for all the children. Most HIV-positive children were diagnosed after lengthy periods of illness and as such, they expressed that they took their medications as prescribed to stay healthy. All of them were in boarding school, and consuming their medications there was an additional burden because they had to hide them from their peers. Most disliked taking the medications, and some thought they interfered with their regular lives (see Table 2, Quote 5). Most HIV-negative children stated that they helped their parents remember to take their medications because the medications had improved their parents’ health after prolonged ill health (see Table 2, Quote 6). Four hoped that their parents would remain healthy, pay their school fees so they could finish school, and have a better life; a few wanted to financially support their parents in the future.

### Sexual awareness

All the children expressed they were not sexually active. Although all HIV-positive children had acquired their illnesses through mother-to-child-transmission, three still had questions about the origin of their illnesses. Some who were teenagers stated they received peer pressure from their HIV-negative peers to engage in the highly prevalent sexual activity present among teenagers. Three of these HIV-positive teenagers expressed that they had questions about their acceptability as relationship partners, use of condoms, marriage, and childbearing options. All three had spoken with healthcare professionals about these issues, but remained dissatisfied with the answers they were provided (see Table 2, Quote 7). Two teenage HIV-negative children with full disclosure suspected that their parents acquired the illness through sexual intercourse but were unable to ask them; however, they expressed that they had spoken with their parents about sexual-related matters. They also agreed that their teenage peers were having sex and expressed a wish for children, especially HIV-negative children, to be taught about the illness so they could be more careful about engaging in sex (see Table 2, Quote 8).

### Coping mechanisms

All children except a preadolescent HIV-positive boy with partial disclosure of his illness, expressed that they had a close trusted person whom they spoke to when feeling down about their circumstances. These persons included their older siblings, cousins, aunts, uncles, grandparents, and friends. All the children including those with partial disclosure expressed that stressful situations, idleness, and periods of unhappiness negatively affected them, causing them to self-withdraw for periods ranging from 30 minutes to two hours. While alone, the children performed a range of activities to help themselves feel better such as thinking about how to improve their lives, praying about their circumstances, reading, watching TV, listening to the radio, and listening, singing, and dancing to music. A few also cried and took naps; one HIV-positive boy who blamed his mother for his illness, hid from her and left the house to play with other children.

HIV-positive children with full disclosure expressed they gained extra support from their HIV-positive peers during support group meetings held at the clinic. They considered these peers as their only true friends and exchanged cell phone numbers so they could keep in touch when back in school. All HIV-positive children expressed a need to be understood, respected, educated on self-care by healthcare professionals, and loved by their parents, relatives, and peers. They especially wanted their HIV-positive peers to care about and look out for each other. Two of them expressed that they did not want to be forced to do chores at home (see Table 2, Quote 9). HIV-negative children expressed a need for healthcare professionals to educate them about the illness and how best to support their parents. Those with full disclosure of their parents’ illnesses expressed a desire to be brought together with other affected children so they could share their experiences and learn from each other (see Table 2, Quote 10).

## Discussion

This study presents results from a small purposively selected and imbalanced sample size of seven HIV-positive and five HIV-negative children; as such the results should be interpreted with caution. Prior studies reporting on the effects of disclosure on children have been conducted with HIV-positive children (Brown et al., 2011; Menon et al., 2007; Petersen et al., 2010; Vaz et al., 2010) and HIV-positive mother-child dyads (Jones et al., 2007; Murphy, Steers & Stritto, 2001; Shafer et al., 2001; Rochat et al., 2014; Rochat, Mkwanazi & Bland, 2013). This study presents the post-disclosure experiences of both HIV-positive and negative children. The results appear to indicate that HIV-positive and negative children have mostly differing post-disclosure experiences revolving around acceptance of illness, high levels of societal misconceptions accompanied by stigma and discrimination, indefinite daily medication consumption necessary for maintenance of good health, and high sexual awareness accompanied by lingering questions about the source of illness, condom use, marriage, and childbearing options. Children used various mechanisms to cope with their circumstances.

HIV-negative children accepted and recovered from disclosure of their parents’ illnesses faster than HIV-positive children recovered from disclosure of their own illnesses. Additionally, some HIV-positive teenagers experienced similar grieving reactions as those seen in teenage HIV-positive Puerto Rican children following disclosure of their illnesses (Blasini et al., 2004). HIV-negative children expressed that they became closer to their parents and were able to speak with them about difficult subjects such as sex. Unlike prior studies that reported increased post-disclosure bonding between parents and their HIV-positive and negative children (Kennedy et al., 2010; Petersen et al., 2010), HIV-positive children in this study did not report increased closeness with their parents. These differences in post-disclosure experiences of HIV-positive and negative children, especially in regards to acceptance of illness and recovery from full disclosure, need to be studied further.

HIV-negative children did not report direct incidences of stigma and discrimination, but all including those with partial disclosure, hid their parents’ illnesses from others. HIV-positive children experienced externalized and internalized stigma through actions shown them by their peers, and extended family and community members. Prior researchers have reported high levels of depression, discrimination, self-stigma, and self-isolation in HIV-positive children after disclosure of their illnesses (Biadgilign et al., 2011; Ishikawa et al., 2010; Petersen et al., 2010; Vaz et al., 2010; Vreeman et al., 2014). From this study’s results, it appears that HIV-negative children also self-isolate themselves when feeling overwhelmed about their circumstances. Given the high prevalence of stigma in Kenya and other nations with high HIV prevalence, it appears that both HIV-positive and negative children may benefit from disclosure services and public education programs aimed at counteracting the high levels of stigma, discrimination, and misconceptions held by community members (Kouyoumdjian, Meyers & Mtshizana, 2005; Murphy & Marelich, 2008; Vaz et al., 2010).

The majority of HIV-negative children wanted their parents to stay healthy and pay their school fees so they could have a better life for themselves in the future. This was unlike findings reported by Kennedy et al. (2010), who found that following disclosure of their parents’ illnesses, some children in that study conducted in the United States, were so distressed that they could not function for a long time. In this study, HIV-positive children disliked taking their medications but appreciated their role in helping them stay healthy. Additional studies are needed to further understand and describe the post-disclosure desires of HIV-negative children in relation to medication consumption by their parents. It also appears that programs and services are needed to assist HIV-positive children to take their medications and maintain adherence.

The 2012 Kenya AIDS Indicator Survey found that despite high awareness of the illness, children were initiating sex as early as 10 years; some had multiple partners with low or no condom use (National AIDS and STI Control Programme Kenya, 2014). Teenage HIV-positive and negative children in this study, confirmed that their peers were having sex. Some HIV-negative children advocated for children to be taught about the illness, and HIV-positive children had many questions about condoms, relationships, marriage, and childbearing. This study’s results appear to indicate that teenage HIV-positive and negative children have a desire to speak and be taught about sexual-related matters. The utility of innovative programs such as Project Mwana (UNICEF, 2014), which uses text messages to disseminate maternal and child health information to program participants, should be investigated in their capability to provide sexual-related information to teenagers. Additionally, it appears that HIV-positive children may benefit from counseling programs and services that regularly apprise them on emerging research findings, such as the use of pre-exposure prophylaxis (PrEP) for conception (Chadwick et al., 2011; Lampe et al., 2011; Savasi et al., 2013; Vernazza et al., 2011) and post-exposure prophylaxis (PEP) for their partners in the event of unprotected sex or if a condom breaks during sexual intercourse (Palmer, 2014; Sultan, Benn & Waters, 2014).

Post-disclosure, prior researchers have called for parents to provide a safe person for their children to speak with (Murphy, 2008; Murphy et al., 2011). In this study, most children had self-identified a person to provide them with social support; HIV-positive children gained additional support within peer support groups. Support groups are known to help HIV-positive children cope with their illnesses (Mawn, 2011; Petersen et al., 2010). Some HIV-negative children in this study wanted to be educated on how to support their parents and also wanted to be brought together with other similarly affected children. Given the scarcity of studies involving HIV-negative children, more studies need to be conducted to understand their post-disclosure needs and if peer support groups and other services (e.g., counseling) may be beneficial for them.

This study’s results appear to support the stress and coping theory (Lazarus, 1993). As seen in prior research (Asander et al., 2009; Kennedy et al., 2010; Murphy et al., 2010; Murphy, 2008; Petersen et al., 2010; Vallerand et al., 2005; Wiener et al., 2007), HIV-positive and negative children in this study experienced both positive and negative effects of disclosure. However, they appeared to be effectively using emotion- and problem-focused behavioral strategies to cope with their ongoing circumstances. When they perceived their levels of stress as increased, all children withdrew to be by themselves and performed positive activities to help themselves feel better. Further testing of the stress and coping theory’s utility in addressing and lessening children’s post-disclosure stressors is warranted, so that programs and services can be created to help HIV-positive and negative children better cope with their circumstances.

This study’s limitations include a small purposively selected sample of mostly teenage children conversant in English who were recruited from an urban area. Due to the small sample size, the results may not be generalizable to other HIV-positive and negative children who have received disclosure of their own and their parents’ illnesses, respectively. Future studies should include larger sample sizes, use local languages, and recruit children of different ages from diverse cultural neighborhoods. The studies should also seek to fill the knowledge gap on the experiences of HIV-positive and negative siblings after they receive full disclosure of their own and their parents’ illnesses within the same household.

## Conclusion

Although from a small sample size, the results of this study appear to indicate that HIV-positive and negative children undergo different experiences after disclosure of their own and their parents illnesses respectively. Many HIV-affected families in highly prevalent nations have both HIV-positive and negative siblings in the household. Until larger studies are conducted, this study’s results should assist healthcare professionals to provide targeted advice to HIV-positive parents who wish to disclose to their children of mixed HIV statuses.

## Supplemental Information

10.7717/peerj.956/supp-1 Table S1 Raw data
