# Supplementary material for: A rapid assessment of post-disclosure experiences of urban HIV-positive and HIV-negative school-aged children in Kenya
Source: PeerJ. 2015 Jun 11;3:e956. doi: 10.7717/peerj.956 (PMC4465943; doi:10.7717/peerj.956)
Supplement: Table S1 [file peerj-03-956-s001.docx]

| Supplemental Table | | |
| --- | --- | --- |
| Children’s Emotional State And Coping Strategies Post-Disclosure | | |
|  | **HIV-Positive**  **Children** | **HIV-Negative**  **Children** |
| Emotional state at time of disclosure | **Positive Feelings:**  Relief from knowing about their illnesses  **Negative Feelings:**  Afraid; Angry; Bad; Confused; Cried; Depressed; Disbelief; Hopelessness; Sadness; Shock; Sorrow; Surprised; Unhappy; Upset; Unlucky; Worry | **Positive Feelings:**  Calm; No big deal; Relief  **Negative Feelings:**  Afraid; Bad; Cried; Hopelessness; Not feeling good; Sad; Shock; Sorrow; Sorry; Surprised; Sympathy for parent; Thoughtful; Unhappy; Worry |
| In the weeks to months after disclosure | **Positive Feelings:**  Acceptance; Feeling better  **Negative Feelings:**  Cried; Depressed; Down; Hopelessness; Low; Sad; Unhappy, Self-hate; Withdrawal; Wonder where illness came from | **Positive Feelings:**  Feeling better; Normal; Okay  **Negative Feelings:**  A little bad |
| Emotions at the time of study participation | **Positive Feelings:**  Comfortable; Happy; Normal; Okay; Hope for cure  **Negative Feelings:**  Crying; Nervousness; Sadness; Stressed; Upset; Worry | **Positive Feelings:**  Feeling better; Hopeful; Increased closeness with parents  **Negative Feelings:**  Hopelessness; Sadness; Sympathy for parent; Upset; Wonder about origin of parents’ illnesses |
| Coping strategies | **HIV-Positive and HIV-Negative Children**  Speaking to a close friend or family member (e.g., older sibling, cousin, aunt, uncle, grandparent)  Dancing  Listening to the radio and/or music  Napping  Praying  Reading  Thinking positive thoughts  Singing  Watching TV  **HIV-Positive Children Only**  Speaking and sharing with peers during support group meetings | |
